# Left Ventricular and Right Ventricular Hypertrophy Modelling to Study PAPP-A-Mediated IGFBP-4 Cleavage-a Mechanism That Regulates IGF Bioavailability in Adult Rats

**DOI:** 10.3390/ijms27062761

**Published:** 2026-03-18

**Authors:** Marina M. Artemieva, Arina V. Makeeva, Daria A. Adasheva, Viacheslav E. Shein, Alexey G. Katrukha, Alexander B. Postnikov, Natalia A. Medvedeva, Daria V. Serebryanaya

**Affiliations:** 1 School of Biology, Lomonosov Moscow State University, 119992 Moscow, Russia; marinka.artemieva@gmail.com (M.M.A.); arinam200231@gmail.com (A.V.M.); nesterova.darya97@gmail.com (D.A.A.); evg.shein2000@mail.ru (V.E.S.); katrukha@mail.ru (A.G.K.); namedved@gmail.com (N.A.M.); 2 HyTest, Ltd., 20520 Turku, Finland; alexander.postnikov@hytest.ru; 3 Institute of Neuroscience and Neurotechnology, Pirogov Russian National Research Medical University, 117997 Moscow, Russia

**Keywords:** cardiac hypertrophy, renovascular hypertension, pulmonary hypertension, PAPP-A-mediated IGFBP-4 proteolysis

## Abstract

Pathological cardiac hypertrophy, a major contributor to heart failure, is characterized by an abnormal increase in the size of atria and ventricles. In the context of ventricular hypertrophy, the right ventricle (RV) exhibits less resistance to hypertrophy than the left one (LV). Insulin-like growth factors (IGF-1 and IGF-2) are critical for cell growth and provide cardioprotective effects. Pregnancy-associated plasma protein-A (PAPP-A) is a protease that cleaves insulin-like growth factor-binding protein-4 (IGFBP-4) and enhances IGF bioavailability. This study investigated PAPP-A-mediated IGFBP-4 proteolysis—one possible mechanism of IGF release regulation in rat models of right ventricular (RVH) and left ventricular (LVH) hypertrophy. RVH was induced with monocrotaline, and LVH via renovascular hypertension (1 Kidney 1 Clip (1K1C) model). Systolic blood pressure was measured using tail-cuff plethysmography. Heart morphometry was used to assess the mass of cardiac chambers. Cardiomyocyte purity was confirmed via troponin I immunocytochemistry. Plasma natriuretic type-B peptide (BNP) and C-terminal IGFBP-4 (CT-IGFBP-4) concentrations were quantified by fluoroimmunoassay. RVH and LVH were successfully modelled, with 1.6-fold and 1.3-fold increases in RV (*p* < 0.0001) and LV masses (*p* < 0.05), respectively. Plasma BNP was 2–3 times higher in LVH versus control rats. Hypertrophied cardiomyocytes secreted significantly more BNP than controls, showing 3.3-fold and 4.1-fold increases in LVH and RVH, respectively. PAPP-A-mediated IGFBP-4 proteolysis was 4-fold higher in RVH compared to control, but unaffected in LVH. These findings suggest that PAPP-A-specific elevation of IGFBP-4 proteolysis occurs predominantly in RVH, suggesting a differential IGF bioavailability in both ventricles and highlighting PAPP-A as a potential target to increase RVH resistance to hypertrophy.

## 1. Introduction

Pathological hypertrophy is an abnormal increase in the size of cardiomyocytes that occurs due to disease or injury, leading to impaired function and potentially heart failure [1]. Unlike physiological hypertrophy, which is induced during physical training or pregnancy, pathological hypertrophy is a maladaptive response to various cardiovascular disease diseases (CVDs), such as hypertension, volume overload or genetic cardiomyopathies [2]. Hypertrophic changes can touch both the ventricles and atria; however, ventricular hypertrophy, which occurs with functional disorders of cardiac activity, such as aortic stenosis, mitral or aortic regurgitation, cardiomyopathies and ischemic cardiac diseases, mostly accompanies CVD development [2]. Ventricular hypertrophy occurs in both the left and right ventricles due to independent causes. Left ventricular hypertrophy (LVH) is caused by cardiovascular pathologies while right ventricular hypertrophy (RVH) is also associated with pulmonary diseases [3].

The IGF (insulin-like growth factor) system plays a significant role in both normal cardiac development and the development of cardiac hypertrophy, both physiological and pathological [4]. Two main ligands of the IGF system—IGF-1 and IGF-2—participate in the development of different hypertrophy types. IGF-1 and its receptor (IGF-1R) are crucial for normal cardiac growth during development and for physiological cardiac hypertrophy. The activation of IGF-1R, through IGF-1 or IGF-2, can promote cardiomyocyte growth and is linked to normal heart development [4,5]. In contrast, IGF-2, particularly in the context of intrauterine growth restriction, has been shown to be involved in the development of pathological cardiac hypertrophy [4,6]. IGF-2R, the IGF-2 receptor, has also been shown to play a role in pathological cardiac hypertrophy, potentially through Gαq signalling [7]. The IGF system is also involved in the overall remodelling of the heart, influencing not just cardiomyocyte size but also other aspects of heart function, including metabolism, autophagy, and apoptosis [4,8]. Because of the involvement of the IGF system in both normal and pathological cardiac growth, it is a potential target for therapeutic interventions related to heart disease [4,9]. For example, IGF-1 supplementation has been explored in cases of IGF-1 deficiency and due to its potential to improve cardiac function [9,10].

The biological effects of IGF-1 and IGF-2 depend directly on their bioavailability to specific receptors [4,11]. IGF-1 and IGF-2 bioavailability, on the one hand, is inhibited via their interaction with IGFBPs 16 (IGF-binding proteins), but on the other hand it is activated by specific proteolytic enzymes that cleave IGFBPs-IGF complex and release IGF, allowing them to bind with their receptors. One well-known regulator of IGF bioavailability is Pregnancy-Associated Plasma Protein A (PAPP-A)—a metalloprotease that specifically cleaves IGFBP-4 between Met135 and Lys136, resulting in the formation of N-terminal and C-terminal proteolytic fragments of IGF-binding protein 4 (NT-IGFBP and CT-IGFBP-4) [4,11]. PAPP-A and IGFBP-4 proteolytic fragments are known as diagnostic and prognostic biomarkers of different pathologies that accompany CVD, like atherosclerosis (PAPP-A), acute coronary syndrome (PAPP-A, IGFBP-4 proteolytic fragments), acute heart failure (IGFBP-4 proteolytic fragments), and cardiac risk assessment (IGFBP-4 proteolytic fragments) [12,13,14]. This may indicate that the activation of PAPP-A-specific IGFBP-4 proteolysis takes place during the pathogenesis of various CVDs. Recently, we showed that IGFBP-4 proteolytic fragments’ formation via PAPP-A is elevated in endothelin-1-hypertrophied rat neonatal cardiomyocytes, suggesting that this mechanism of IGF bioavailability increase might be enhanced during hypertrophy development at the molecular and cellular levels [15].

It was previously demonstrated that mechanisms regulating IGF bioavailability may participate in both LVH and RVH development; however, the main studies on IGF’s role in pathological cardiac development focused on LVH [16,17]. Moreover, elevated PAPP-A-mediated IGFBP-4 cleavage has only been demonstrated in a primary cardiomyocyte culture model with a hypertrophic phenotype induced by endothelin-1, which does not provide complete information on the pathogenesis of cardiac hypertrophy at the physiological level [15], and there have been no studies utilizing in vivo hypertrophy models. Thus, in the present study we investigate PAPP-A-dependent IGFBP-4 proteolysis as a potential mechanism of IGF release using both RVH and LVH modelling by applying a novel approach that integrates physiological and cellular studies.

## 2. Results

### 2.1. Pulmonary Hypertension Modelling and Characterization

**Figure 1:**
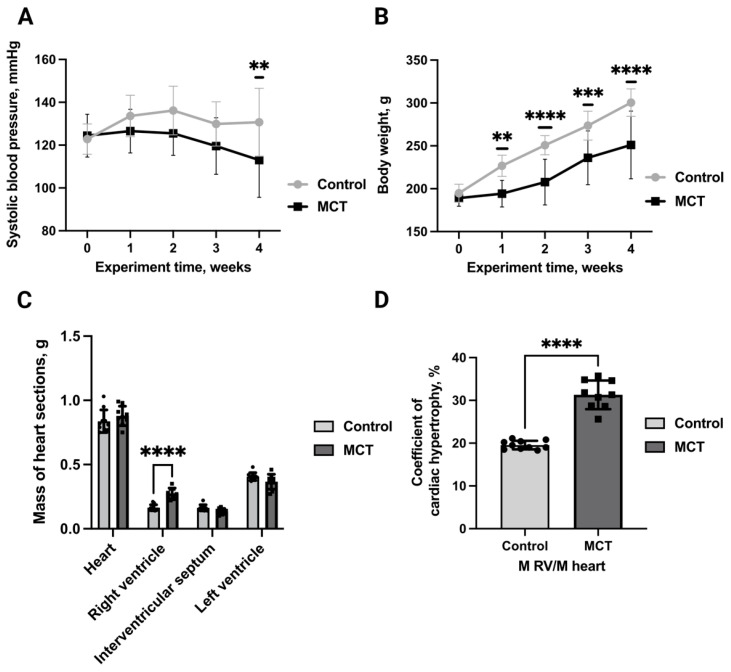
(**A**) Dynamics of systolic blood pressure change in MCT rats. **—statistically significant differences in MCT group (n = 9) compared to the control group (n = 10), *p* < 0.01. (**B**) Dynamics of body weight change in MCT rats. **—statistically significant differences in MCT group compared to the control group, *p* < 0.01. ***—statistically significant differences in MCT group (n = 9) compared to the control group (n = 10), *p* < 0.001. ****—statistically significant differences in MCT group (n = 9) compared to the control group (n = 10) *p* < 0.0001. (**C**) Mass of heart sections of RVH rats. ****—statistically significant differences in MCT group (n = 9) compared to the control group (n = 10), *p* < 0.0001. (**D**) Right ventricular hypertrophy severity ratios in MCT rats. M, mass expressed in grams; LV—left ventricle. ****—statistically significant differences in MCT group (n = 9) compared to the control group (n = 10), *p* < 0.0001. Control—control rats; MCT—monocrotaline rats.

First, we modelled RVH utilizing monocrotaline-induced pulmonary hypertension. We chose this model because it is well-described and reliable. Pulmonary hypertension was induced in 20 rats via monocrotaline injection, with 20 controls. No mortality occurred in either group. The dynamics of systolic blood pressure over the 4-week experimental period are presented in Figure 1A. Baseline systolic blood pressure values were comparable between groups (control: 122.9 ± 7 mmHg; MCT: 124.4 ± 10 mmHg). By week 4, the MCT group exhibited a statistically significant reduction in systolic blood pressure compared to controls, reaching final values of 113.0 ± 18 mmHg versus 130.7 ± 16 mmHg, respectively. The development of hypertension was associated with significantly greater weight gain by week 4 post-injection (Figure 1B) in control rats (300.5 vs. 194.8 g at baseline) compared to MCT-treated animals (189 vs. 251 g at baseline). Cardiac analysis (Figure 1C) revealed statistically significant differences, specifically in right ventricular mass, between groups (0.27 g for MCT vs. 0.16 g for control), with the MCT group exhibiting a 1.6-fold increase in the right ventricular hypertrophy index (RVMH) compared to controls (Figure 1D), suggesting successful RVH modelling.

### 2.2. Renovascular Hypertension Modelling and Characterization

**Figure 2:**
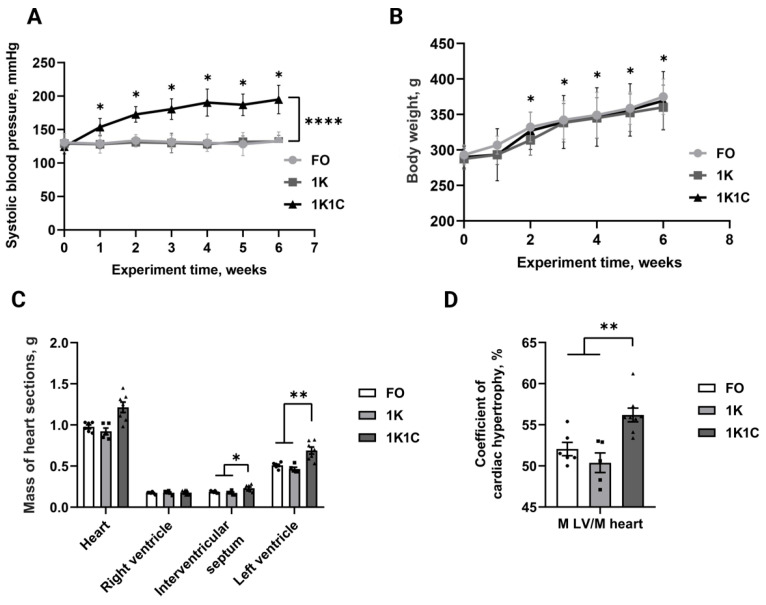
(**A**) Dynamics of systolic blood pressure change in 1K1C rats compared to the control groups. *—statistically significant differences in the 1K1C group relative to baseline blood pressure in this group, *p* < 0.0001. ****—statistically significant differences in group 1K1C (n = 19) compared to 1K (n = 16) and FO (n = 19) groups, *p* < 0.0001. (**B**) Dynamics of body weight change in RVH rats. *—statistically significant differences from the zero point in all experimental groups *p* < 0.0001. (**C**) Mass of heart sections of RVH rats. *—statistically significant differences in group 1K1C (n = 7) compared to groups 1K (n = 6) and FO (n = 5), *p* < 0.05; **—statistically significant differences in group 1K1C (n = 7) compared to 1K (n = 6) and FO (n = 5) groups, *p* < 0.001. 1K1C—one kidney, one clip rats; 1K—one kidney rats; FO—false-operated rats. (**D**) Left ventricular hypertrophy severity ratios in RVH rats. M, mass expressed in grams; LV—left ventricle. **—statistically significant differences in group 1K1C compared to 1K and FO groups, *p* < 0.01. 1K1C—one kidney, one clip rats; 1K—one kidney rats; FO—false-operated rats.

To model LVH, renovascular hypertension (1K1C-1 kidney 1 clip) was generated. Following renovascular hypertension induction, we obtained 32 1K1C (1 kidney 1 clip), 16 1K (1 kidney), and 20 FO (false-operated) rats. Mortality rates were 34.4% (1K1C), 17.6% (1K), and 5% (FO). Blood pressure analyses revealed a sustained 1.5-fold increase in SBP (Figure 2A) in 1K1C (n = 19) rats by week 6 post-operation (194.9 vs. 124.5 mmHg at baseline; *p* < 0.0001). Control groups (1K, n = 16, 132.2 vs. 129.1 mmHg at baseline, and (FO, n = 19, 132.5 vs. 130.8 mmHg at baseline) showed no significant blood pressure changes during this period (*p* > 0.05).

No significant differences in body weight (Figure 2B) were observed between groups (*p* > 0.05), with all rats demonstrating weight gain during the six-week postoperative period: 1K1C (369.2 by week 6 vs. 289.8 g at baseline; *p* < 0.0001), 1K (360 by week 6 vs. 287.3 g at baseline; *p* < 0.0001), and FO (293 by week 6 vs. 374.8 g at baseline; *p* < 0.0001). Cardiac analysis revealed a 30% increase in left ventricular mass (1K1C: 0.65 g, n = 5; 1K: 0.47 g, n = 3; FO: 0.52 g, n = 4; *p* < 0.05) and a 20% increase in interventricular septal mass (1K1C: 0.23 g, n = 5; 1K: 0.17 g, n = 3; FO: 0.19 g, n = 4; *p* < 0.05) in the experimental group compared with controls (Figure 2C). Right ventricular mass did not differ significantly between groups (*p* > 0.5). The left ventricular hypertrophy index (LVMH) relative to heart mass (Figure 2D) was also significantly higher in the experimental group (1K1C: 55.5%, n = 5; 1K: 50.6%, n = 3; FO: 51.7%, n = 4; *p* < 0.01). Thus, LVH was also effectively induced in adult rats.

### 2.3. BNP Quantification in Rat Blood Serum

**Figure 3:**
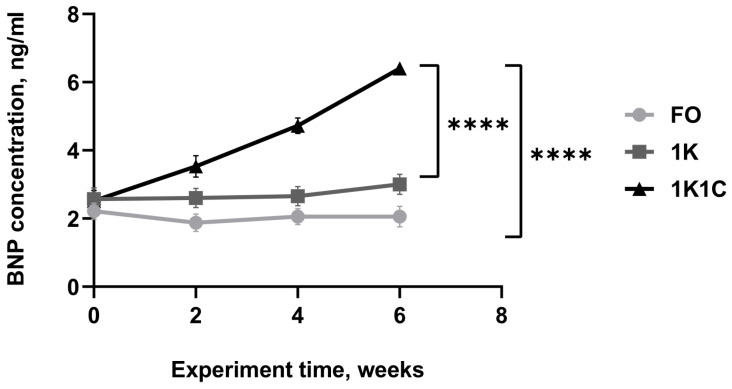
Dynamics of BNP concentration change in blood serum of LVH rats ****—statistically significant differences between experimental group 1K1C (n = 12) and control groups 1K (n = 14) and FO (n = 13) at week 6 post-hypertension induction, *p* < 0.0001. 1K1C—one kidney, one clip rats; 1K—one kidney rats; FO—false-operated rats.

We also analyzed hypertrophy development at both the physiological and cellular levels. We measured the concentration of B-type natriuretic peptide, which is well known as a biomarker of hypertrophy and pathogenesis of HF. By week 6 post-operation, blood serum BNP concentration (Figure 3) in 1K1C rats (n = 12) increased 2.6-fold (6.4 vs. 2.5 ng/mL at baseline), reaching levels 3.0-fold higher (2.1 ng/mL) than in FO rats (n = 13) and 2.1-fold higher (3.0 ng/mL) than in 1K rats (n = 14). No significant differences were found between the control groups (*p* > 0.05).

### 2.4. Cardiomyocyte Cultures’ Immunochemical Characterization

**Figure 4:**
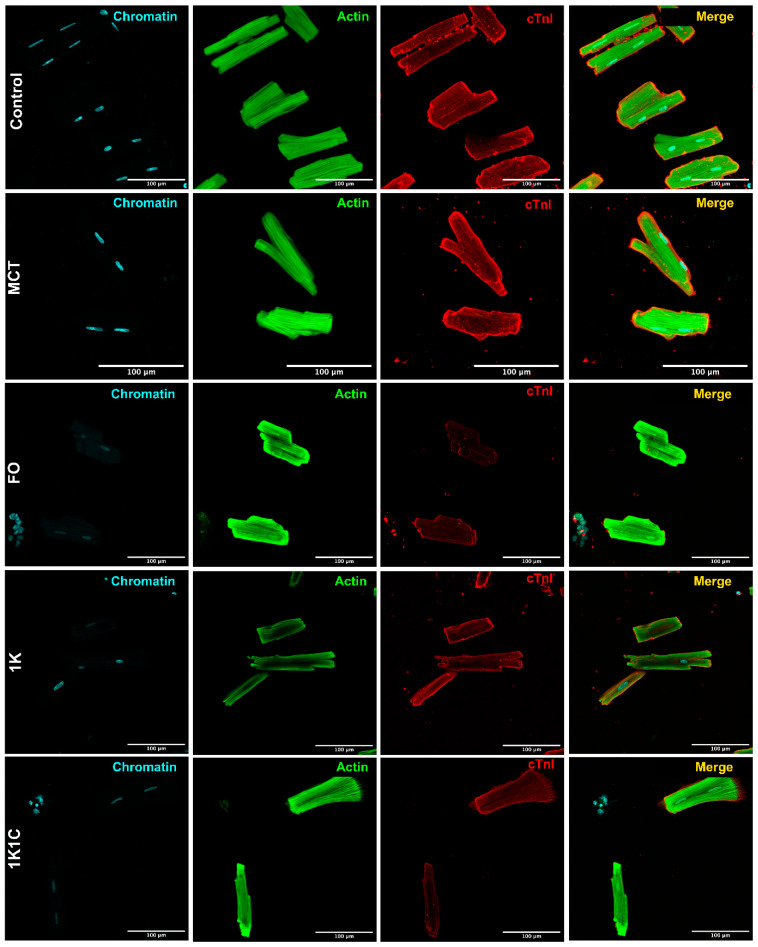
Immunostaining of adult ventricular cardiomyocyte cultures. Cells were immunostained with DAPI (Chromatin, blue), Alexa-488-phalloidin (Actin, green), and cardiac troponin I antibodies with Alexa-555 secondaries (cTnI, red). This staining pattern was consistent across all adult rat ventricular cardiomyocyte cultures studied (control, MCT, FO, 1K, and 1K1C groups). Control—control rats, MCT—monocrotaline rats, 1K1C—one kidney, one clip rats; 1K—one kidney rats; FO—false-operated rats.

After RVH and LVH modelling was performed, the primary cardiomyocyte cultures were obtained from the ventricles of normal and hypertrophied animals. Culture purity was assessed by quantifying cardiomyocyte content based on the immunostaining results (Figure 4). The total culture area (100%) was determined using phalloidin-stained actin (green channel). Cardiomyocyte-specific area was calculated as the percentage of cardiac troponin I-positive cells (red channel) relative to total cells. Adult rat cardiomyocytes exhibited characteristic cylindrical morphology and striation. The uniform troponin I expression (detected in all cells) and consistent morphology confirmed 100% culture purity.

### 2.5. Quantification of Cardiomyocyte Area in the MCT-Induced Hypertrophy Model

**Figure 5:**
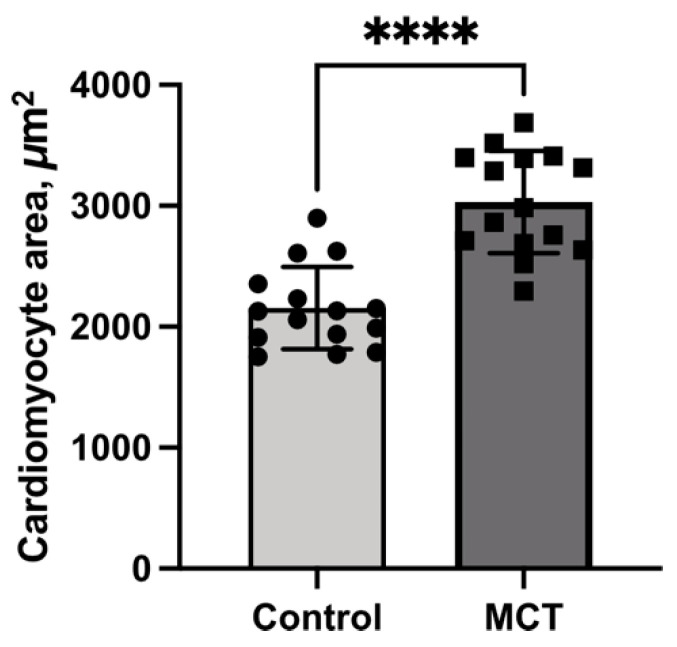
Effect of MCT treatment on cardiomyocyte size. Cardiomyocyte area was measured in the control (n = 15) and MCT (n = 15) groups. Data are presented as mean ± standard deviation. Statistical analysis was performed using an unpaired *t*-test; **** *p* < 0.0001 vs. control.

Following hypertrophy induction, cardiomyocytes were isolated from the hearts of adult control and experimental rats, as detailed in the Methods. The resulting cultures were examined microscopically, and the cardiomyocyte area was measured using ImageJ 2.0 software. As shown in Figure 5, the cardiomyocyte area was 1.3-fold greater in the MCT group than in the controls. These findings confirm the development of myocardium hypertrophy and demonstrate the persistence of hypertrophic changes in isolated cardiomyocyte culture.

### 2.6. BNP Quantification in Conditioned Medium of Control and Hypertrophied Cardiomyocytes

**Figure 6:**
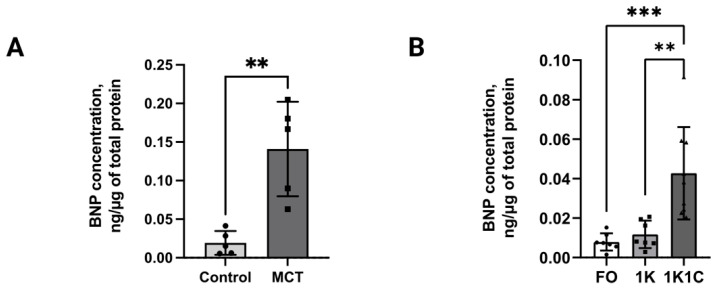
(**A**) BNP concentration in the conditioned medium of primary adult rat ventricular cardiomyocytes from MCT rats. **—statistically significant differences between experimental group MCT (n = 5) and control group (n = 5), *p* < 0.01. (**B**) BNP concentration in the conditioned medium of primary adult rat ventricular cardiomyocytes from RVH rats. **—statistically significant differences between experimental group 1K1C (n = 9) and control groups 1K (n = 7) and FO (n = 7), *p* < 0.005. ***—statistically significant differences between experimental group 1K1C (n = 9) and control groups 1K (n = 7) and FO (n = 7), *p* < 0.001. Control—control rats; MCT—monocrotaline rats; 1K1C—one kidney, one clip rats; 1K—one kidney rats; FO—false-operated rats.

To check whether obtained cardiomyocytes retained the hypertrophied phenotype or not, the BNP concentration was also measured in their conditioned media. Mann–Whitney test (Figure 6A) of fluoroimmunoassay data demonstrated a 7.3-fold higher BNP concentration (0.14 ng/µg of total protein, n = 5, *p* < 0.01) in adult ventricular cardiomyocyte-conditioned medium from MCT rats versus control group (0.02 ng/µg of total protein, n = 5, *p* < 0.0). One-way ANOVA (Figure 6B) of fluoroimmunoassay data demonstrated a 4.7-fold higher BNP concentration (0.04 ng/µg, *p* < 0.001) in adult ventricular cardiomyocyte-conditioned medium from 1K1C rats (n = 9) versus 1K (n = 7, 0.01 ng/µg) and FO (n = 7, 0.01 ng/µg) groups, which showed equivalent BNP levels (*p* > 0.05). These findings suggest that cardiomyocytes preserve the hypertrophied pattern after purification, opening up the possibility of studying hypertrophy at both the physiological and cellular level within a single animal.

### 2.7. CT-IGFBP-4 Quantification in Conditioned Medium of Control and Hypertrophied Cardiomyocytes

**Figure 7:**
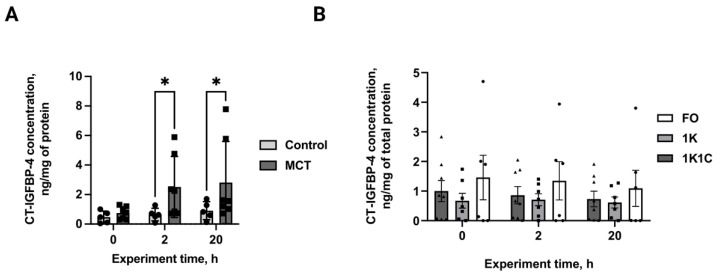
(**A**) CT-IGFBP-4 concentration in the conditioned medium of primary adult rat ventricular cardiomyocytes from MCT rats. *—statistically significant differences between experimental group MCT (n = 5) and control group (n = 5), *p* < 0.05. (**B**) CT-IGFBP-4 concentration in the conditioned medium of primary adult rat ventricular cardiomyocytes from the 1K1C (n = 8), 1K (n = 7) and FO (n = 6) groups. Control—control rats; MCT—monocrotaline rats; 1K1C—one kidney, one clip rats; 1K—one kidney rats; FO—false-operated rats.

PAPP-A-specific IGFBP-4 proteolysis was assessed in the conditioned media of hypertrophied and normal cardiomyocytes. Paired *t*-tests revealed a 3-fold elevation in CT-IGFBP-4 concentration in monocrotaline-treated adult rat ventricular cardiomyocytes versus controls (2.51 vs. 0.65 ng/mg at 2 h; 2.81 vs. 0.91 ng/mg at 24 h; *p* < 0.01, n = 5 both), with sustained proteolytic differences throughout incubation (Figure 7A). No significant differences were observed between groups in RVH rats (Figure 7B, *p* > 0.05, n = 6, 7, 8 for FO, 1K, 1K1C respectively). Thus, PAPP-A-specific IGFBP-4-cleavage is elevated in RV, but not in LV cardiomyocytes, suggesting different patterns of IGF bioavailability regulation in both ventricles.

## 3. Discussion

It is generally accepted that RV and LV have different anatomical structures—RV has thin walls and a crescent shape while LV possesses thicker walls and a conic shape [18]. The lower pulmonary arterial pressure and pulmonary vascular resistance correspond to 20% and 10% of systemic arterial pressure and systemic vascular resistance, respectively, leading to lower oxygen consumption by the RV compared to LV [18,19]. This makes the RV less vulnerable to myocardial ischemia. However, the RV is highly susceptible to acute increases in afterload, unlike the LV. The LV and RV also have enormous differences in the shape and duration of their action potential [20]. In the context of hypertrophy, compensatory remodelling is restricted in the RV compared to the LV. Pressure overload in the RV in pulmonary artery banding (PAB) models leads to higher mortality than pressure overload in the LV by aortic constriction. PAB models also produce more elevated hypoxia in the RV after surgery, with lower capillary density and less ischemia [21]. RV is also more susceptible to fibrotic remodelling during pressure overload than LV [22]. Thus, these studies hint that RV is more susceptible to hypertrophic changes than LV. 

In our study we applied an approach that combines in vivo RVH and LVH modelling followed by in vitro cardiomyocyte isolation to study PAPP-A-mediated IGFBP-4 proteolysis—one of the mechanisms that regulate IGF bioavailability [23]. This approach allows us to study hypertrophy at both the physiological and cellular levels. The combined model design included two main stages. First, RVH and LVH modelling was performed in adult rats using monocrotaline (MCT) and renovascular hypertension induction, respectively. Both models are widely utilized in physiological studies; however, they have advantages and disadvantages. The MCT model is quite easy to handle and can be used to model RVH in 6 weeks; however, MCT is toxic and might induce other pathologies, like hepatic veno-occlusive disease. The model of renovascular hypertension stimulation is known for its high reproducibility of renin-dependent hypertension. Its disadvantages include the invasiveness of surgery, the potential for high mortality, and its limited emulation of human chronic atherosclerotic disease. Our choice of these models was based on their reproducibility and easy handling compared to the other models. In the present study, we managed to successfully model RVH and LVH in rats using the aforementioned methodologies. The obtained hypertrophy indexes that were calculated and the increase in BNP concentration indicate that hypertrophy was successfully induced using both techniques. In our study, BNP was chosen as a biomarker for hypertrophy. Although its association with LV pressure overload is prominent, its utility as a biomarker for RVH has also been extensively documented in the literature [24,25,26,27]. Accordingly, we utilized BNP levels as an indicator of RVH as well. The second step of this combined methodology is the isolation of cardiomyocytes from hypertrophied rat hearts. We demonstrated that all of them express a cardiac isoform of troponin I-cardiac protein that is specific for cardiomyocytes and showed that the obtained cultures retained elevated BNP expression levels that reflect the maintenance of the hypertrophied phenotype. We chose BNP measurement as the key test to analyze hypertrophy because it indicates the physiological state of the cell and demonstrates the hypertrophied phenotype. Together with the specific increase in cardiomyocyte size, it provides strong evidence of a retained hypertrophied phenotype in cardiomyocytes after purification. Thus, we developed a model that combined in vivo and in vitro properties and allowed us to analyze both the physiological and cellular effects of hypertrophic pathogenesis within a single animal. Finally, we measured PAPP-A-specific IGFBP-4 proteolysis level in conditioned media of control and hypertrophied cardiomyocytes and found that, in cardiomyocytes obtained from MCT rats (RVH), IGFBP-4 proteolytic cleavage by PAPP-A is increased, while in cardiomyocytes obtained from rats with renovascular hypertension (LVH), no significant changes were observed compared to the control groups. PAPP-A-specific IGFBP-4 proteolysis was evaluated by CT-IGFBP-4 measurements, although NT-IGFBP-4 led to similar results. This data is in agreement with our previous results showing that PAPP-A-mediated IGFBP-4 proteolysis is increased in endothelin-1-induced hypertrophied primary cardiomyocytes since endothelin-1-activated signalling is associated with RVH development [15,28]. Consequently, the differences in PAPP-A-mediated IGFBP-4 proteolysis could be linked not only to chamber-specific characteristics but also to the degree of cellular stress and disease progression. Nevertheless, the literature indicates that both models share common features, such as inflammation and ROS production, with no significant disparities reported [29]. Given that the degree of hypertrophy in our study was relatively similar between MCT and renovascular models (1.4-fold vs. 1.2-fold, respectively), we conclude that the observed elevation in PAPP-A-specific activity towards IGFBP-4 is more likely attributable to the specific mechanisms of RVH [30].

This is probably connected to the less expressed compensatory reaction in RV compared to the LV–pathological remodelling of cardiomyocytes, which may trigger cardioprotective adaptive mechanisms including an increase in IGF release via PAPP-A-mediated IGFBP-4 cleavage. Our study also raised a question regarding whether PAPP-A-mediated IGFBP-4 proteolysis increased because of the elevated protein expression or the augmentation of its enzyme activity. However, PAPP-A protein expression is rather difficult to measure since it is secreted outside of the cell, and could not be detected by immunohistochemistry or Western blotting. mRNA quantification by qPCR does not completely reflect the quantity of the enzymatically active PAPP-A and ELISA requires antibodies specific to rat PAPP-A. The limitations of our study included the inability to measure PAPP-A using the “sandwich”-type fluoroimmune assay since the available antibodies were specific to human PAPP-A and did not recognize the rat protein. Nevertheless, the results of the present study open up the possibility of further investigation into the regulation of IGF bioavailability by PAPP-A in the RV and LV and an exploration of its downstream signalling pathways.

## 4. Materials and Methods

### 4.1. Physiological Methods

#### 4.1.1. Pulmonary Hypertension Modelling

**Figure 8:**
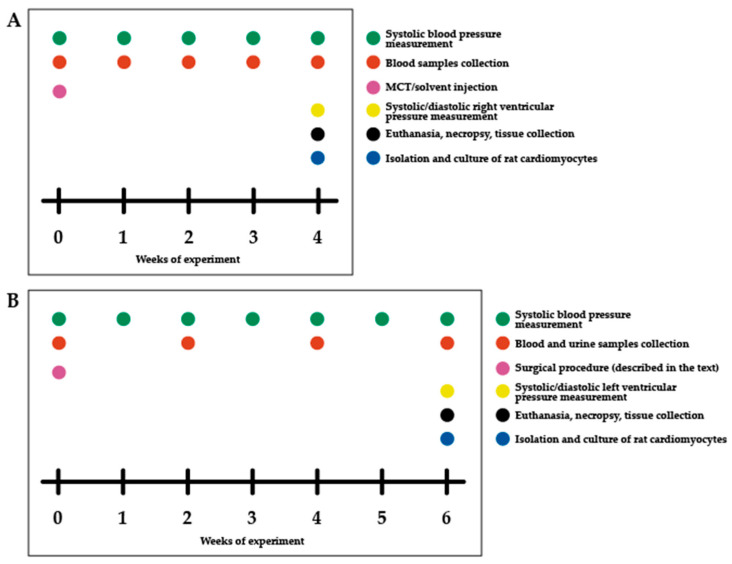
(**A**) Scheme of the experiment with pulmonary hypertension modelling. (**B**) Scheme of the experiment with renovascular hypertension modelling.

The pulmonary hypertension model (Figure 8A) was modelled in Wistar male rats (180–200 g) via monocrotaline (MCT), as previously described [31]. The animals were divided into control and MCT groups by simple randomisation. MCT was dissolved in 60% ethanol (20 mg/mL) and administered subcutaneously (60 mg/kg) at three points between the shoulder blades and the left and right legs. Control rats received an equivalent volume of 60% ethanol. All animals were housed in a vivarium with ad libitum access to food and water for four weeks post-injection.

#### 4.1.2. Renovascular Hypertension Modelling

Renovascular hypertension (Figure 8B) was modelled in Wistar male rats (250–300 g) as described [32]. The animals were randomly divided into three groups. Randomization was performed within the blocks such that the mean systolic blood pressure was approximately equal across all animal model groups. 1K1C (one kidney, one clip): the right kidney was excised, and a silver clip (0.26 mm diameter) was placed on the left renal artery. 1K (one kidney): the right kidney was removed, leaving the left kidney intact. False-operated (FO): rats underwent a mock surgical procedure. Post-surgery, rats were individually housed with ad libitum access to food and water. For three days, they received intraperitoneal ciprofloxacin (Ciprinol^®^, Krka, Slovenia; 5 mg/kg, 2 mL/day). Body temperature was monitored, and glucose/saline was administered as needed.

#### 4.1.3. Body Weighing and Systolic Blood Pressure Measurement

Body weight for both RVH-rats and MCT-rats was recorded before surgery and weekly thereafter. Systolic blood pressure (SBP) was also measured weekly by using tail-cuff plethysmography (Lgraph2 2.34.10 software, L-CARD, Moscow, Russia, with a strain gauge) (Figures S2–S4). Preoperative SBP (measured 2–3 days before surgery or injection) served as the baseline. Before each measurement, rats underwent 10 min warming for vasodilation and were restrained in a plexiglass restrainer. Values were averaged from five to six measurements per rat.

#### 4.1.4. Blood Samples Collection

Blood samples from RVH-rats were collected every two weeks for six weeks post-surgery. Blood samples from MCT-rats were also collected every week for four weeks post-injection. Rats were briefly warmed and immobilized, and 1 mL of blood from the tail vein was collected via syringe and transferred to a tube. Samples were allowed to clot at room temperature (30 min), refrigerated at 4 °C (30 min), then centrifuged at 500× *g* to isolate serum. All the samples were stored at −20 °C.

#### 4.1.5. Organ Morphometry

Six weeks after hypertension induction, organ morphometry was performed on a subset of the animals. Rats were anesthetized with urethane (1.2 g/kg) and decapitated. The heart, lungs, and kidneys were excised, and their masses were measured, including the atria, left ventricle (LV), right ventricle (RV), and interventricular septum. We also calculated hypertrophy indexes (Figure S1). After euthanasia, the rat’s heart was removed, washed in saline buffer, and dried on a napkin. All surrounding vessels and tissue were removed, the heart was weighed, and the atria were carefully removed, and the right and left ventricles and septum were weighed separately. The hypertrophy index for each cardiac section was calculated as the ratio of the mass of the heart section to the total heart weight.

### 4.2. Cell Isolation and Characterization Methods

#### 4.2.1. Primary Cardiomyocytes Isolation

Primary cardiomyocytes were isolated from adult rats using a modified Alam et al. protocol [33]. Rats received an intraperitoneal injection of heparin (200 µL, 5000 IU) 10 min before decapitation. Hearts were excised, rinsed in Krebs–Henseleit solution (NaCl 118 mM, KCl 4.8 mM, HEPES 25 mM, MgSO_4_ 1.25 mM, K_2_HPO_4_ 1.25 mM, glucose 11 mM, taurine 5 mM, pH 7.4), and cannulated via the aorta for Langendorff perfusion. Cardiomyocytes were isolated from RV or LV, as described in Alam et al., with centrifugation adjusted to 100× *g* (instead of 20× *g*). RV and LV were dissected after Langendorf perfusion. For enhanced procedural clarity and ease, we hung the heart on cannula in the following direction: the ventral part was oriented to the scientist, with the right ventricular on the left side, and the left ventricular on the right side. We also utilized ligatures of distinct colours to differentiate the right and left sides. Cells were plated on culture dishes and confocal chambers were pre-coated with fibronectin (40 µg/mL, overnight).

#### 4.2.2. Cultures’ Immunochemical Characterization

Cultures were fixed with 4% PFA and characterized by immunostaining according to the protocol [34]. Cells were immunostained using cTnI-specific (cardiac troponin I) antibodies (MF4cc; Hytest, Turku, Finland). Cells were co-stained with phalloidin (Alexa-488; Hytest, Turku, Finland) and DAPI. Secondary polyclonal antibodies against mouse Fc-fragments were conjugated with Alexa-555. Preparations were visualized using an Olympus FV-300 inverted fluorescence microscope (Olympus Corp., Tokyo, Japan).

#### 4.2.3. Cardiomyocyte Area Quantification

To assess cardiomyocyte hypertrophy, cell surface area was measured using immunocytochemically stained isolated ventricular cardiomyocytes imaged by confocal microscopy. Images were captured at 60× magnification to ensure a detailed visualization of the cytoskeleton for the precise delineation of cell borders. Surface area measurements were performed using ImageJ 2.0g (Fiji). After calibrating the scale (pixels to microns), individual cardiomyocytes exhibiting a regular, cylindrical morphology with clearly defined edges were manually traced using the polygon tool to define the region of interest. The area was then calculated using the ‘Analyze–Measure’ command. Cellular debris and damaged cells were excluded from the analysis. The culture preparation consisted exclusively of cardiomyocytes, eliminating the risk of inadvertently measuring non-myocyte cells. Due to the limited cell adherence following immunocytochemical staining, images were acquired from five distinct fields per sample, with each field containing 2–5 cells. Consequently, a total of 15 cardiomyocytes were measured per experimental group (approximately 3 cells per field × 5 fields). The mean cell surface area was then calculated for each group.

#### 4.2.4. IGFBP-4 Proteolysis Induction

IGFBP-4 proteolysis was quantified using a modified Laursen et al. method [35]. The substrate consisted of recombinant human IGFBP-4, IGF-II, and CaCl_2_ in the TBS buffer, which was pre-incubated for 30 min at room temperature. This mixture was added to the cardiomyocyte culture medium and incubated at 37 °C (5% CO_2_) for 1–24 h. Reactions were terminated with EDTA (5 mM final concentration). CT-IGFBP-4 levels were measured by fluorescent sandwich immunoassay. The results (ng/mL) were normalized to a total protein concentration determined by Bradford assay.

### 4.3. Biochemical Methods

#### 4.3.1. BNP Quantification via Fluoroimmunoassay

A three-step sandwich fluoroimmunoassay (FIA) was used to quantify B-type natriuretic peptide (BNP) in the blood serum of RVH rats (10× diluted) and undiluted adult ventricular cardiomyocyte-conditioned medium for both MCT and RVH rats, following the established protocol [36]. Monoclonal antibodies (24C5 or 50E1cc, 10 µg/mL in PBS; Hytest, Finland) served as the primary (capture) antibodies, while europium–chelate-conjugated monoclonal antibodies (Ab-BNP2Eu* or 130ccEu*, 4 µg/mL in assay buffer) functioned as secondary (detector) antibodies. Fluorescence intensity was measured after adding LANFIA detection solution using a Victor 1420 Multilabel Counter (PerkinElmer Inc., Waltham, MA, USA) with the Europium protocol.

#### 4.3.2. CT-IGFBP-4 Quantification via Fluoroimmunoassay

CT-IGFBP-4 concentration in adult ventricular cardiomyocyte conditioned medium was quantified using a neoepitope-specific fluorescent immunoassay (IBP182-IBP163Eu*, Hytest, Finland) according to the established protocol [34]. Microplates were coated with capture antibodies (IBP182, 10 µg/mL in PBS) for 1 h, followed by incubation with conditioned medium samples and detector antibodies (IBP163Eu*, 2 µg/mL in assay buffer). Fluorescence was measured as presented above.

### 4.4. Statistical Analysis

Statistical analyses were conducted using GraphPad Prism (v 10.3, GraphPad Software). The normality of data distribution was evaluated using the Shapiro–Wilk test. For data conforming to a normal distribution, statistical analyses were conducted employing Student’s *t*-test and one-way analysis of variance (ANOVA) with Šidák’s correction, followed by Tukey’s post hoc test, or two-way ANOVA with Tukey’s post hoc test for multiple comparisons. The Mann–Whitney U-test was used for non-normally distributed data.

For physiological measurements (blood pressure, body weight, and morphometry), each experimental replicate corresponded to one rat, with values averaged within groups. Biochemical assays were performed in duplicate for each sample, with results averaged per animal and then across groups. Statistical significance was defined as a two-tailed *p* < 0.05.

## Data Availability

Data supporting the findings of this study are available from the corresponding author upon request.

## Supplementary Materials

The following supporting information can be downloaded at: https://www.mdpi.com/article/10.3390/ijms27062761/s1.

## References

[1] Kavazis A.N. (2015). Pathological vs. physiological cardiac hypertrophy. J. Physiol..

[2] Nakamura M., Sadoshima J. (2018). Mechanisms of physiological and pathological cardiac hypertrophy. Nat. Rev. Cardiol..

[3] Bhattacharya P.T., Shams P., Ellison M.B. (2025). Right Ventricular Hypertrophy.

[4] Adasheva D.A., Serebryanaya D.V. (2024). IGF Signaling in the Heart in Health and Disease. Biochemistry.

[5] Panic A., Stanimirovic J., Obradovic M., Sudar-Milovanovic E., Isenovic E. (2018). IGF-1 regulates cardiac hypertrophy and inos expression in obese male rats through ERK1/2 signaling pathway. Atherosclerosis.

[6] Wang K.C.W., Brooks D.A., Botting K.J., Morrison J.L. (2012). IGF-2R-Mediated Signaling Results in Hypertrophy of Cultured Cardiomyocytes from Fetal Sheep1. Biol. Reprod..

[7] Wang K.C.W., Tosh D.N., Zhang S., McMillen I.C., Duffield J.A., Brooks D.A., Morrison J.L. (2015). IGF-2R-Gαq signaling and cardiac hypertrophy in the low-birth-weight lamb. Am. J. Physiol.-Regul. Integr. Comp. Physiol..

[8] Annunziata M., Granata R., Ghigo E. (2011). The IGF system. Acta Diabetol..

[9] Huang K.W., Wang I.H., Fu P., Krum H., Bach L.A., Wang B.H. (2021). Insulin-like growth factor-1 directly affects cardiac cellular remodelling via distinct pathways. IJC Heart Vasc..

[10] Hu Y., Jiang Y., Duan L., Yang S., Tuniyazi S., Zou J., Ma R., Muhemaitibieke G., Amuti X., Guo Y.Y. (2024). IGF-1 levels in the general population, heart failure patients, and individuals with acromegaly: Differences and projections from meta-analyses—A dual perspective. Front. Cardiovasc. Med..

[11] Dya G.A., Lebedeva O.S., Gushchevarov D.A., Volovikov E.A., Belikova L.D., Kopylova I.V., Postnikov A.B., Artemieva M.M., Medvedeva N.A., Lagarkova M.A. (2024). Specific cleavage of IGFBP-4 by papp-a in nervous tissue. Biochem. Biophys. Res. Commun..

[12] Bayes-Genis A., Conover C.A., Overgaard M.T., Bailey K.R., Christiansen M., Holmes D.R.J., Virmani R., Oxvig C., Schwartz R.S. (2001). Pregnancy-associated plasma protein A as a marker of acute coronary syndromes. N. Engl. J. Med..

[13] Funayama A., Shishido T., Netsu S., Ishino M., Sasaki T., Katoh S., Takahashi H., Arimoto T., Miyamoto T., Nitobe J. (2011). Serum pregnancy-associated plasma protein a in patients with heart failure. J. Card. Fail..

[14] Postnikov A.B., Smolyanova T.I., Kharitonov A.V., Serebryanaya D., Kozlovsky S., Tryshina Y., Malanicev R., Arutyunov A., Murakami M., Apple F. (2012). N-terminal and C-terminal fragments of IGFBP-4 as novel biomarkers for short-term risk assessment of major adverse cardiac events in patients presenting with ischemia. Clin. Biochem..

[15] Serebryanaya D.V., Adasheva D.A., Konev A.A., Artemieva M.M., Katrukha I.A., Postnikov A.B., Medvedeva N.A., Katrukha A.G. (2021). IGFBP-4 Proteolysis by PAPP-A in a Primary Culture of Rat Neonatal Cardiomyocytes under Normal and Hypertrophic Conditions. Biochem. Mosc..

[16] Donohue T.J., Dworkin L.D., Lango M.N., Fliegner K., Lango R.P., Benstein J.A., Slater W.R., Catanese V.M. (1994). Induction of myocardial insulin-like growth factor-I gene expression in left ventricular hypertrophy. Circulation.

[17] Duerr R.L., Huang S., Miraliakbar H.R., Clark R., Chien K.R., Ross J. (1995). Insulin-like growth factor-1 enhances ventricular hypertrophy and function during the onset of experimental cardiac failure. J. Clin. Investig..

[18] Bernal-Ramirez J., Díaz-Vesga M.C., Talamilla M., Méndez A., Quiroga C., Garza-Cervantes J.A., Lázaro-Alfaro A., Jerjes-Sanchez C., Henríquez M., García-Rivas G. (2021). Exploring Functional Differences Between the Right and Left Ventricles to Better Understand Right Ventricular Dysfunction. Oxidative Med. Cell. Longev..

[19] Crystal G.J., Pagel P.S. (2018). Right Ventricular Perfusion: Physiology and Clinical Implications. Anesthesiology.

[20] Calloe K., Aistrup G.L., Di Diego J.M., Goodrow R.J., Treat J.A., Cordeiro J.M. (2018). Interventricular differences in sodium current and its potential role in Brugada syndrome. Physiol. Rep..

[21] Ikeda S., Satoh K., Kikuchi N., Miyata S., Suzuki K., Omura J., Shimizu T., Kobayashi K., Kobayashi K., Fukumoto Y. (2014). Crucial role of rho-kinase in pressure overload-induced right ventricular hypertrophy and dysfunction in mice. Arterioscler. Thromb. Vasc. Biol..

[22] Minegishi S., Kitahori K., Murakami A., Ono M. (2011). Mechanism of pressure-overload right ventricular hypertrophy in infant rabbits. Int. Heart J..

[23] Makeeva A.V., Artemieva M.M., Adasheva D.A., Shein V.E., Medvedeva N.A., Serebryanaya D.V. (2025). A Combination of In Vitro and In Vivo Approaches to Studying the Mechanisms of Myocardial Hypertrophy Development in Adult Rats with Renovascular Hypertension. J. Evol. Biochem. Physiol..

[24] Noritoshi N., Toshio N., Yoshiaki O., Uematsu M., Satoh T., Kyotani S., Kuribayashi S., Hamada S., Kakishita M., Nakanishi N. (1998). Plasma Brain Natriuretic Peptide Levels Increase in Proportion to the Extent of Right Ventricular Dysfunction in Pulmonary Hypertension. J. Am. Coll. Cardiol..

[25] Casserly B., Klinger J.R. (2009). Brain natriuretic peptide in pulmonary arterial hypertension: Biomarker and potential therapeutic agent. Drug Des. Devel Ther..

[26] Yap L.B. (2004). B-type natriuretic Peptide and the right heart. Heart Fail. Rev..

[27] Cepkova M., Kapur V., Ren X., Quinn T., Zhuo H., Foster E., Matthay M.A., Liu K.D. (2011). Clinical significance of elevated B-type natriuretic peptide in patients with acute lung injury with or without right ventricular dilatation: An observational cohort study. Ann. Intensive Care.

[28] Dobrian A.D., Davies M.J., Schriver S.D., Lauterio T.J., Prewitt R.L. (2001). Oxidative stress in a rat model of obesity-induced hypertension. Hypertension.

[29] Bhat M., Zaid M., Singh S., Gill K., Tantray J., Sharma R.K., Singh M., Mishra A., Singh R.P., Sahu S.K. (2023). A current review on animal models of anti-hypertensive drugs screening. Health Sci. Rev..

[30] Nagendran J., Sutendra G., Paterson I., Champion H.C., Webster L., Chiu B., Haromy A., Rebeyka I.M., Ross D.B., Michelakis E.D. (2013). Endothelin Axis Is Upregulated in Human and Rat Right Ventricular Hypertrophy. Circ. Res..

[31] Hojda S.-E., Chis I.C., Mîrza T.-V., Clichici S. (2024). Monocrotaline-induced pulmonary arterial hypertension: The benefic effects of magnesium sulfate, Rosuvastatin and Sildenafil. Med. Pharm. Rep..

[32] Ilatovskaya M.E., Pozdnyov V.F., Andreev-Andrievskyy A.A., Medvedeva N.A. (2012). Blockade of endothelin-1 synthesis enhances the development of renovascular hypertension in rat experiments. Russ. J. Physiol..

[33] Alam P., Maliken B.D., Ivey M.J., Jones S.M., Kanisicak O. (2020). Isolation, Transfection, and Long-Term Culture of Adult Mouse and Rat Cardiomyocytes. J. Vis. Exp..

[34] Adasheva D.A., Lebedeva O.S., Goliusova D.V., Postnikov A.B., Teriakova M.V., Kopylova I.V., Lagarkova M.A., Katrukha A.G., Serebryanaya D.V. (2023). PAPP-A-Specific IGFBP-4 Proteolysis in Human Induced Pluripotent Stem Cell-Derived Cardiomyocytes. Int. J. Mol. Sci..

[35] Laursen L.S., Overgaard M.T., Nielsen C.G., Boldt H.B., Hopmann K.H., Conover C.A., Sottrup-Jensen L., Giudice L.C., Oxvig C. (2002). Substrate specificity of the metalloproteinase pregnancy-associated plasma protein-A (PAPP-A) assessed by mutagenesis and analysis of synthetic peptides: Substrate residues distant from the scissile bond are critical for proteolysis. Biochem. J..

[36] Tamm N.N., Seferian K.R., Semenov A.G., Mukharyamova K.S., Koshkina E.V., Krasnoselsky M.I., Postnikov A.B., Serebryanaya D.V., Apple F.S., Murakami M.M. (2008). Novel immunoassay for quantification of brain natriuretic peptide and its precursor in human blood. Clin. Chem..

