# Supplementary figures and images for: Left Ventricular and Right Ventricular Hypertrophy Modelling to Study PAPP-A-Mediated IGFBP-4 Cleavage-a Mechanism That Regulates IGF Bioavailability in Adult Rats
Source: Int J Mol Sci. 2026 Mar 18;27(6):2761. doi: 10.3390/ijms27062761 (PMC13026115; doi:10.3390/ijms27062761)

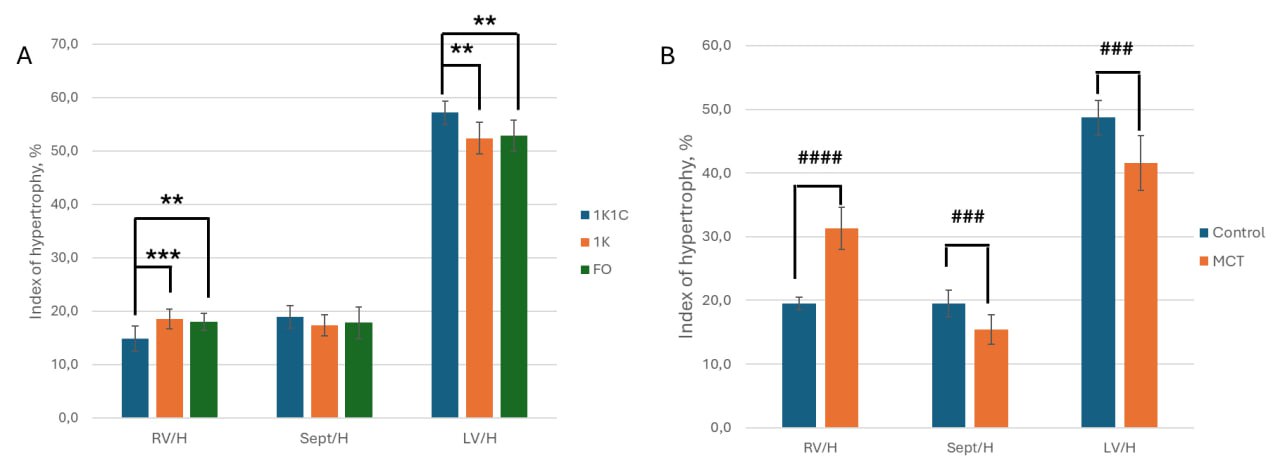

Supplement: Supplementary file 1 [file ijms-27-02761-s001.zip › Figure S1.jpg]

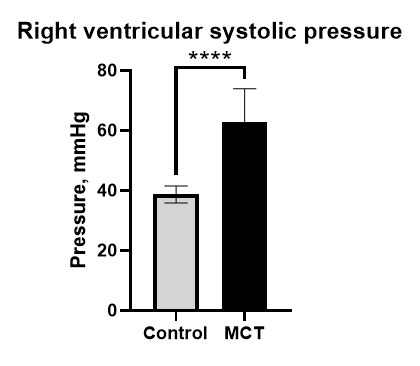

Supplement: Supplementary file 1 [file ijms-27-02761-s001.zip › Figure S2.jpg]

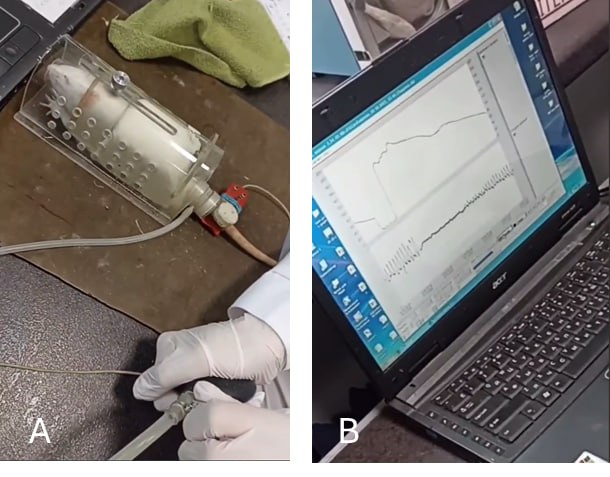

Supplement: Supplementary file 1 [file ijms-27-02761-s001.zip › Figure S3.jpg]

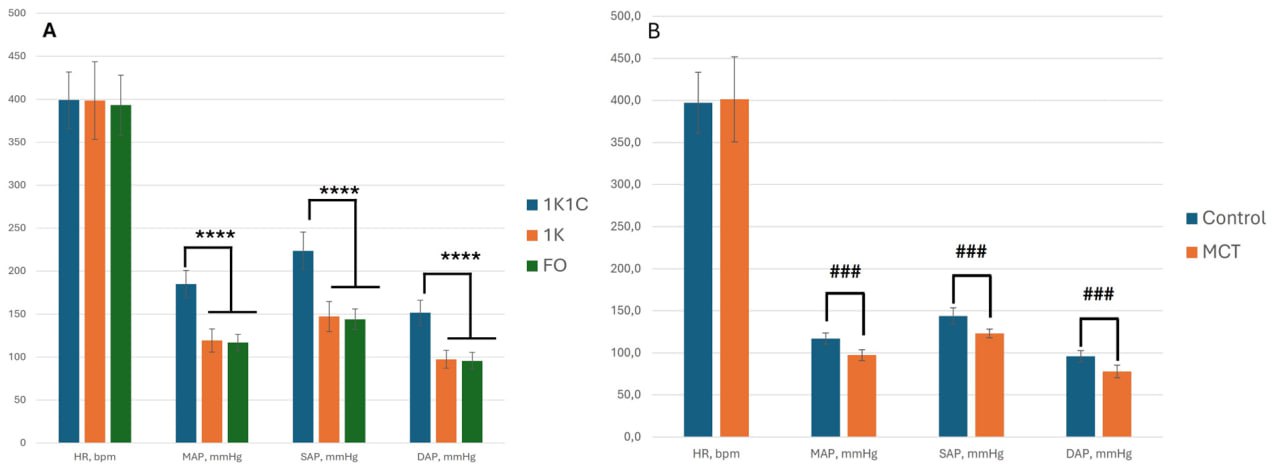

Supplement: Supplementary file 1 [file ijms-27-02761-s001.zip › Figure S4.jpg]
